# Site-Specific Nested Integration of Tn*1806* into ICE*Sa*2603-Family Integrative and Conjugative Elements in *Streptococcus agalactiae*

**DOI:** 10.3390/microorganisms14020375

**Published:** 2026-02-05

**Authors:** Sida Yi, Xing Xu, Liufan Yin, Zhichun He, Xueliang Wang

**Affiliations:** 1 Department of Molecular Biology, Shanghai Center for Clinical Laboratory, Shanghai 200126, China; ysd951114@163.com (S.Y.);; 2 Department of Quality Control Material R&D, Shanghai Center for Clinical Laboratory, Shanghai 200126, China

**Keywords:** integrative conjugative elements, *Streptococcus agalactiae*, composite ICEs, conjugation transfer, ICE*Sa*2603, Tn*1806*, horizontal gene transfer

## Abstract

Composite integrative and conjugative elements (ICEs) frequently mediate the co-transfer of multiple antibiotic resistance genes during horizontal gene transfer, but their formation mechanisms remain unclear. This study investigated the site-specific integration of Tn*1806* into ICE*Sa*2603-family ICEs in *Streptococcus agalactiae* by conjugation experiments. PCR screening of 161 *S. agalactiae* clinical isolates identified potential Tn*1806*-like ICE carriers; whole-genome sequencing was performed to further characterize the macrolide-resistance isolates from this group. PCR detection resulted in 24 carrying Tn*1806*-like ICEs being found, five of which were macrolide-resistant. Genomic analysis for these five revealed distinct Tn*1806*-like ICEs (ICE*Sag*16, ICE*Sag*57, ICE*Sag*139, ICE*Sag*167, and ICE*Sag*220), three of which were found nested within another ICE (ICE*Spy*009, an ICE*Sa*2603-family ICE). Conjugation experiments confirmed ICE*Sag*167 could integrate into the *snf2* (methyltransferase containing a SNF2 helicase domain) of ICE*Spy*009 in recipient cells, generating a composite ICE. Re-conjugation verified the transferability of composite ICE at low frequencies (8.63 × 10^−8^), during which some nested ICE*Sag*167 were excised and transferred independently. This work provides first experimental evidence supporting Tn*1806* nesting within another ICE as a mechanism for resistance accumulation and mobile element evolution in *S. agalactiae*. The spread of such composite ICEs may confer multiple forms of resistance to new hosts, challenging infection treatment and raising public health concerns.

## 1. Introduction

Integrative and conjugative elements (ICEs), a class of self-transmissible mobile genetic elements (MGEs), reside in bacterial chromosomes and mediate functional gene unit dissemination between different hosts [1,2]. Under specific conditions, ICEs are excised from chromosomes by encoded site-specific integrases and transferred to the recipient bacteria through the conjugation machinery, conferring novel phenotypes, such as pathogenicity and antibiotic resistance, to the new hosts [3,4]. Whole-genome sequencing has identified numerous ICEs carrying antibiotic resistance genes and facilitating their horizontal dissemination [5,6,7,8,9]. Consequently, ICEs play a critical role in driving the evolution of bacterial antimicrobial resistance.

*Streptococcus agalactiae* (group B *Streptococcus*), a leading cause of neonatal sepsis and meningitis, is a significant pathogen among pregnant women, the elderly, and immunocompromised individuals [10]. In this clinically important pathogen, ICEs have been identified as primary vectors for the dissemination of multidrug resistance [11]. These elements are not only widely prevalent but also frequently harbor clusters of resistance genes, conferring resistance to aminoglycosides, macrolides, lincosamides, tetracyclines, and even oxazolidinones [12,13,14]. In 2019, the U.S. Centers for Disease Control and Prevention (CDC) classified clindamycin-resistant *S. agalactiae* as a “concerning threat” among 21 antibiotic resistance threats [15].

Horizontal gene transfer mediated by ICEs represents a major driver of microbial evolution, enabling microorganisms to rapidly acquire new genes and phenotypes. An analysis of over 1000 genomes identified 335 putative ICEs and 180 conjugative plasmids, indicating that ICEs are present in most bacterial clades and are likely more common than conjugative plasmids [16]. However, ICEs are not static genetic entities. They can exhibit considerable genetic diversity through chimeric recombination events, which may alter their core characteristics. This dynamic, mosaic nature complicates efforts to monitor and control the antibiotic resistance associated with ICEs [1,17]. In some cases, ICEs act as vectors by incorporating exogenous ICEs/mobile elements or integrate into other ICEs/mobile elements: ICE*Sag*TR7 forms a “Matryoshka doll”-like structure, created when a Tn*1806*-like ICE carrying the *erm*(TR) gene into an ICE*Sa*2603-family ICE backbone (ICE*Sde*3396) [18]. ICE*Sag*236 resulted from a recombination between ICE*Spn*529IQ and ICE*Spy*009, with deletions in both components, and its transfer mediates the spread of macrolide, lincosamide, and streptogramin B (MLSB) and chloramphenicol resistance [19]. Although composite ICEs like ICE*Sag*TR7 have been documented, the mechanism enabling Tn*1806*—an element carrying *erm*(TR) first identified in *Streptococcus pneumoniae* [20]—to integrate into a resident ICE and confer additional resistance, remains unconfirmed experimentally. This gap in understanding may stem from the unconfirmed independent transferability of Tn*1806*, leading to its characterization primarily as a component of composite ICE, rather than as a distinct mobile entity.

Recently, a novel Tn*1806*-like ICE, designated ICE*San*95_*hsdM*, was identified in *S. anginosus* [21]. This element carries an *erm*(B)-carrying fragment shares a high degree of identity with Tn*6002*, providing the first experimental confirmation of the transferability for this ICE type. Comparative analysis revealed that ICE*San*95_*hsdM* possesses a putative integration target within *snf2* (encoding the sucrose non-fermenting 2 protein), that is highly conserved in the backbone of ICESa2603-family ICEs [13]. Therefore, we hypothesized that large composite ICEs (e.g., ICE*Sag*TR7) arise from the site-specific integration of Tn*1806*-like ICEs into the *snf2* gene within ICE*Sa*2603, acquiring resistance determinants to form composite structures.

This study experimentally demonstrates that composite ICE derived from ICE*Sa*2603-family ICEs are formed when a Tn*1806*-like ICE integrates site-specifically into the conserved *snf2* gene of the ICE*Sa*2603 backbone. This integration physical nests one ICE within another. Consequently, multiple resistance genes accumulate within a single mobile element. The resulting composite ICE exhibits greater flexibility and adaptability, which may enhance the versatility of horizontal gene transfer in bacterial populations.

## 2. Materials and Methods

### 2.1. Bacterial Strains and Susceptibility Tests

The 161 clinical *S. agalactiae* isolates and ATCC13813 used in this study were preserved at Shanghai Center for Clinical Laboratory. All strains were collected from routine laboratory procedures and utilized for scientific research purposes. For enrichment, *S. agalactiae* strain was cultured in Todd–Hewitt Broth (THB; Qingdao Hope Bio-Technology Co., Ltd., Qingdao, China) supplemented with 5% calf serum (Tianhang Biological Products Co., Ltd., Huzhou, China) at 37 °C with shaking at 150 rpm under 10% CO_2_ for 12–24 h. Antimicrobial susceptibility testing was performed on Mueller–Hinton (MH) agar (Qingdao Hope Bio-Technology Co., Ltd.) supplemented with 5% horse serum (YINUO Co., Ltd., Hohhot, China), with incubation at 37 °C under 10% CO_2_ for 48 h. This study involved no personal information or clinical trials. In accordance with our institutional guidelines and relevant regulations, ethical approval was therefore not required for the use of these bacterial strains.

Broth microdilution method was used to perform routine antimicrobial susceptibility testing on *S. agalactiae* isolates against clinical common antibiotics, including erythromycin, clindamycin, levofloxacin, chloramphenicol, penicillin, amoxicillin, and linezolid. These antibiotics were purchased from Sigma Chemical Co., St. Louis, MO, USA. The *S. agalactiae* ATCC 13813 was used as the quality control strain. The minimum inhibitory concentration (MIC) breakpoints complied with the standards of Clinical and Laboratory Standards Institute (CLSI M100-ed34, 2024) [22].

### 2.2. PCR Amplifications

PCR primers targeting conserved genes (encoding integrases, relaxases, and T4SS components) were designed based on three homologous ICEs: Tn*1806* from *S. pneumoniae* and ICE*San*95 from *S. anginosus* (Table S1). Detection of all three conserved genes in a clinical strain suggested at least one putative Tn*1806*-like ICE.

Putative transconjugants colonies were confirmed by PCR. A colony was confirmed as a genuine transconjugant—a recipient that had acquired Tn*1806*—only if PCR detected both the donor-specific *erm*(TR) gene from Tn*1806* and the recipient-specific tyrosine integrase gene from the resident ICE*Spy*009.

PCR primers targeting distinct ICE integration sites were designed to detect different ICE states through the pairwise combinations P1-P2, P3-P4, P1-P4, and P2-P3, as described previously [23]. Primer pairs P1-P2 and P3-P4 detect ICE*Sag*167 integration at the *snf2*, where P2 and P3 are outward-facing primers within end of ICE*Sag*167, while P1 and P4 are inward-facing primers within *snf2* gene. P1*_hsdM_*-P2 and P3-P4*_hsdM_* were used to detect the integration of ICE*Sag*167 at *SE864_01385* (*hsdM*, encoding methyltransferase with a YeeA domain) site; P1*_MTase_*-P2 and P3-P4*_MTase_* were used to detect the integration of ICE*Sag*167 at an Eco57I restriction-modification methylase protein, *SE864_06140* (MTase). To conclusively verify the results, the PCR products indicative of ICE integration and circularization was confirmed by Sanger sequencing.

### 2.3. Determination of ICE Integration Sites

To screen the integration site of ICE*Sag*167 after conjugation, PCR primers were designed based on the known target sites of Tn*1806*-like ICEs (Tn*1806*, ICE*Sag*167, ICE*Sag*16, ICE*San*95_*hsdM*, ICE*Sag*139). First, the complete open reading frames (ORFs) of ICE target genes were retrieved from the NCBI database: Tn*1806* (AP018937, *hsdM*), ICE*Sag*167 (CP029749, *DLM82_06795*), ICE*Sag*16 (CP051004, *GRB95_01380*), ICE*San*95 (CP053789, *GE023_005635*), and ICE*Sag*139 (CP053789, *GE023_005635*). Primers were then designed to flank the predicted *attB* within each target ORF. All primer sequences are listed in Table S1.

### 2.4. Conjugation Transfer Experiments

The selection of recipient strains was based on two criteria: (1) displaying the complementary resistance phenotype to the donor, and (2) containing at least one integration target site for ICEs. Prior to this study, a clinica*l S. agalactiae* strain, *Sag*R31 (Accession No. CP138369) had been whole-genome sequenced. This strain was found to carry an ICE*Sa*2603 family ICE, highly similar to ICE*Spy*009 at 3′ *rplL* gene. It was resistant to tetracycline but susceptible to clindamycin, a profile opposite to that of the donor strain *S. agalactiae Sag*167, which was resistant to erythromycin and clindamycin but susceptible to tetracycline. Both *Sag*R31 and *Sag*167 were susceptible to levofloxacin. Therefore, *Sag*R31 was selected as the recipient for the first round of conjugation. In the re-transfer experiment, the recipient was selected from 161 clinical *S. agalactiae* isolates. These were screened by PCR to confirm an unoccupied *rplL* site. Strain *Sag*RR40, which had an unoccupied *rplL* detected by other elements, was susceptible to erythromycin and clindamycin but resistant to levofloxacin, and was chosen as the recipient for the second round of conjugation.

The conjugation transfer experiment was performed as described previously [12,21]. Donors and recipients were cultured to the logarithmic phase and adjusted to OD_600_ = 0.5. Take 100 μL of donor culture to serially dilute and plate on medium for colony counting. Then donors and recipients were mixed at a 1:10 ratio, spread on nitrocellulose membrane-overlaid agar, and incubated for 4 h. An amount of 10 mg/mL of DNase I was added to eliminate the potential effects of DNA transformation [24]. The DNase I was purchased from YEASEN Biotechnology (Shanghai) Co., Ltd. After the incubation, the mixed bacteria were scraped from membranes and cultured in the medium containing 50 μg/mL erythromycin, 50 μg/mL clindamycin, and 40 μg/mL tetracycline and/or 20 μg/mL levofloxacin. Selected transconjugants were further verified by PCR and sequencing.

The conjugation frequency (*F*) was calculated as *F* = *Nt*/*N*d, where *Nt* and *N*d represent the number of transconjugants and donor cells, respectively. To determine the donor count, a 100 μL aliquot from a 50 mL donor culture was serially diluted 10-fold in PBS six to ten times. After mixing, 100 μL of the appropriate dilution was spread onto counting plates and incubated for one to two days, with colony counts between 30 and 300 considered valid. For transconjugant enumeration, cells were harvested from the nitrocellulose membrane after mating using a sterile swab and resuspended. This suspension was then plated onto transconjugant selective medium either undiluted or following a 10-fold or 100-fold dilution.

### 2.5. DNA Sequencing and Comparison Analysis

Based on previous reports, Tn*1806-like* ICEs frequently carry macrolide resistance genes such as *erm*(B), *erm*(TR), or *mef*(E) [20,21,25]. Therefore, we selected macrolide-resistant isolates from the 24 *S*. *agalactiae* harboring Tn*1806*-like ICEs for further sequencing (Table 1). This selection targeted strains more likely to carry these relevant resistance genes within their ICEs.

Conjugation transfer of ICEs was verified through next-generation sequencing (NGS) of recipient strains and corresponding transconjugants. Genomic DNA extraction employed the QIAGEN Midi Kit (Qiagen, Hilden, Germany), and whole-genome sequencing services were provided by BGI Genomics using the Illumina HiSeq X platform (San Diego, CA, USA). Sequencing data were quality trimmed, and Illumina Nextera indexes were removed using Trimmomatic v0.39.82 [26]. The high-quality reads were de novo assembled using Spades v3.9.034 [27]. Genome annotation was performed using online tool rast (https://rast.nmpdr.org) (accessed on 15 May 2025) [28]. Genomes sequences produced in our study were deposited in NCBI, following accession numbers: recipient *Sag*R31 (CP138369), transconjugant *Sag*R31_TC1 (CP138371), *Sag*R31_TC2 (JAYLLK000000000), *Sag*R31_TC3 (CP138364), *Sag*R31_TC4 (CP138367), re-recipient *Sag*RR40 (CP138370), re-transconjugants *Sag*RR40_TC1 (CP138368), and *Sag*RR40_TC2 (CP139638) (all of which are listed in Table 2).

## 3. Results

### 3.1. Early Characteristics of Tn1806-Positive Isolates

Among 161 *S. agalactiae* strains, 24 were preliminarily identified as potential Tn*1806*-like ICE carriers. Five strains (*Sag*16, *Sag*57, *Sag*139, *Sag*167, and *Sag*220), which exhibited high levels of erythromycin resistance (minimum inhibitory concentration > 128 mg/L), were selected for whole-genome sequencing (Appendix A Table A1).

**Figure 1:**
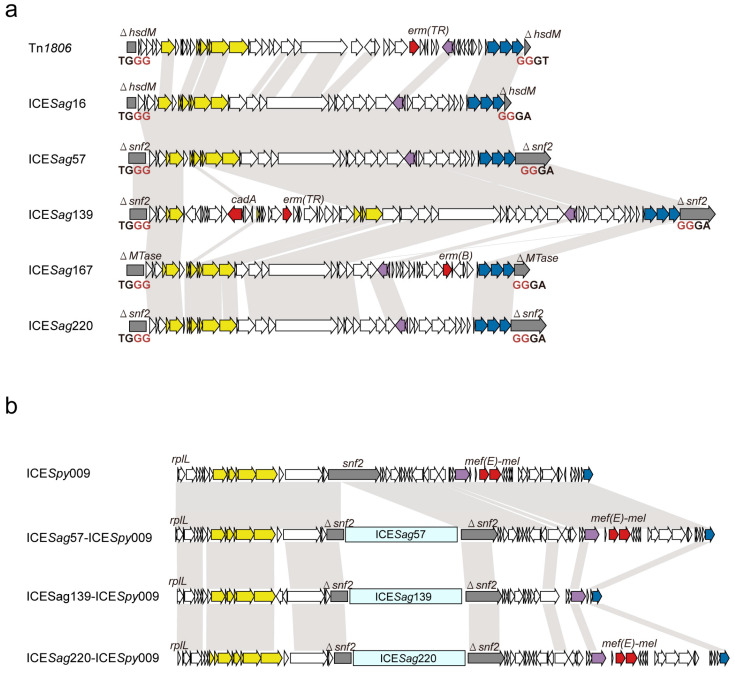
Schematic, but to scale, representation comparing putative ICEs identified in this study with known ICEs. (**a**) Comparison against five newly identified Tn*1806*-like ICEs (designated ICE*Sag*16, ICE*Sag*57, ICE*Sag*139, ICE*Sag*167, and ICE*Sag*220) with Tn*1806* (EF469826). Triangular symbol (Δ) represents truncated ORFs that were inserted by ICEs; areas shaded gray represent regions of identity between 70 and 100%. Integrase genes are shown as blue arrows, relaxase genes as purple arrows, T4SSs and T4CPs components as yellow arrows, antibiotics and heavy metal resistance genes as red arrows, ICE insertion associated genes as light gray arrows, and variable genes as white arrows. The characteristic flanking direct repeats (GG-GG) are highlighted by red. (**b**) Comparison of three composite ICEs (designated ICE*Sag*57-ICE*Spy*009, ICE*Sag*139-ICE*Spy*009, and ICE*Sag*220-ICE*Spy*009) with the known ICE ICE*Spy*009 (KU056701). The light blue rectangle represents Tn*1806*-like ICEs (ICE*Sag*57, ICE*Sag*139, and ICE*Sag*220) that are embedded within *snf2* of the ICE framework.

Whole-genome sequencing confirmed the presence of complete Tn*1806*-like ICEs in all five strains. Table 1 summarizes their genetic features, antimicrobial resistance profiles, and the genomic location of each Tn*1806*-like ICE. Among these, *Sag*167 carried *erm*(B), *Sag*139 carried *erm*(TR), and *Sag*16, *Sag*57, and *Sag*220 harbored the *mel-mef*(E) macrolide resistance gene cluster. However, macrolide resistance genes were located within the Tn1806-like ICEs only in the strains *Sag*167 [*erm*(B)] and *Sag*139 [*erm*(TR)] (Figure 1a).

### 3.2. Characteristics and Genetic Context of Tn1806-like ICEs

Whole-genome sequencing identified the five complete Tn*1806*-like ICEs of 49.005–71.946 kb size (designated ICE*Sag*16, ICE*Sag*57, ICE*Sag*139, ICE*Sag*167, and ICE*Sag*220, respectively, based on the strain abbreviation [*Sag*] and the number of hosts) in five clinical isolates (Figure 1, Table 1). These ICEs encode highly conserved triple serine integrases (>70% amino acid identity) with a known ICE Tn*1806* [20], but each exhibits a distinct chromosomal attachment site (*attB*). ICE*Sag*16 targeted *attB* within the *hsdM* genes, which encodes a methyltransferase with a YeeA domain. ICE*Sag*57, ICE*Sag*139, and ICE*Sag*220 integrated into the *snf2* gene, encoding a methyltransferase protein containing an SNF2 helicase domain, while ICE*Sag*167 is integrated within a gene encoding a protein with an Eco57I restriction modification methylase domain (WP_224219255.1), a methyltransferase abbreviated herein as MTase (Figure 1a). A shared feature was the presence of conserved 4 bp direct repeat sequences (5′-TGGG-GGGA-3′ or 5′-TGGG-GGGT-3′) flanking the five ICE boundaries. Integration into these *attB* sites resulted in the splitting of the target genes into two truncated open reading frames flanking the ICE boundaries.

Analysis of the resistance determinants showed that ICE*Sag*167 carried *erm*(B) conferring macrolide–lincosamide–streptogramin B (MLS_B_) resistance, while ICE*Sag*139 harbored *erm*(TR) (another MLS_B_ determinant) along with *cadA*, a heavy metal resistance gene typically associated with the ICE*Sa*2603 family [29]. Further examination of genomic contexts revealed that ICE*Sag*57, ICE*Sag*139, and ICE*Sag*220 were nested within another ICE exhibiting high similarity to ICE*Spy*009, an ICE*Sa*2603 family member from *S. pyogenes* (Figure 1b) [30]. Given that the *snf2* gene serves as a conserved integration site in ICE*Sa*2603-family ICEs, we hypothesized that Tn*1806*-like ICEs may have undergone specific integration into ICE*Spy*009 backbone to generate composite ICEs.

### 3.3. ICESag167 Integration into ICESpy009 via Conjugation Transfer Experiments

**Figure 2:**
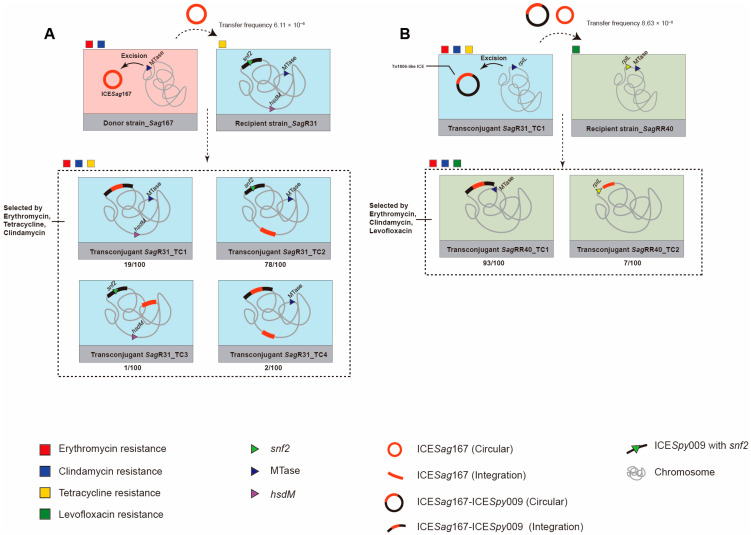
Diagram of the conjugation transfer and re-conjugation transfer experiments. (**A**) experimental workflow for conjugative transfer of ICE*Sag*167 from donor *S. agalactiae Sag*167 to recipient *Sag*R31 and generated four types of transconjugants. Transconjugant were selected by antibiotics erythromycin, tetracycline and clindamycin. Four types of transconjugants: 19/100 *Sag*R31_TC1, 78/100 *Sag*R31_TC2, 1/100 *Sag*R31_TC3, and 2/100 *Sag*R31_TC4. (**B**) re-conjugative transfer of ICE*Sag*167–IC*Spy*009 or ICE*Sag*167 from *Sag*R31_TC1 to recipient *Sag*RR40 and generated two types of transconjugants. Transconjugant were selected by antibiotics erythromycin, clindamycin, and levofloxacin. Two types of transconjugants: 93/100 *Sag*RR40_TC1 and 7/100 *Sag*R40_TC2.

To test the hypothesis, experiments were performed using *Sag*167 (ERY^R^, CLI^R^, TET^S^, and LEV^S^) as the donor strain, which carries ICE*Sag*167 harboring the MLS_B_ resistance gene *erm*(B). *Sag*R31 (ERY^S^, CLI^S^, TET^R^, and LEV^S^) was selected as the recipient strain, as its genome had been fully sequenced prior to this study (Figure 2A, Table 2). Genomic analysis of the recipient *Sag*R31 (accession no. CP138369) revealed three putative integration sites for ICE*Sag*167: *SE864_06335* (*snf2*), *SE864_01385* (*hsdM*), and *SE864_06140* (MTase). Importantly, an ICE*Spy*009-like ICE (located at genomic coordinates 1,220,484–1,277,017) containing the *snf2* gene was identified at 3′ *rplL*. Conjugation experiments successfully generated transconjugants at a frequency of 6.11 × 10^−6^. Subsequent antimicrobial susceptibility testing confirmed that the putative transconjugants exhibited high-level MLS_B_ and tetracycline resistance, consistent with the phenotype of both donor and recipient strains.

To determine the integration sites of ICE*Sag*167 in the recipient, 100 transconjugant colonies were randomly selected from the selective medium and analyzed by PCR. This analysis revealed that 19 of the 100 transconjugants, designated *Sag*R31_TC1, carried a single copy of ICE*Sag*167 integrated at the *SE864_06335* (*snf2*) locus, which aligns with the primary objective of this study. Whole-genome sequencing further confirmed the presence of a 106.508 kb composite ICE, formed by ICE*Sag*167 and the resident ICE*Spy*009 and designated ICE*Sag*167–ICE*Spy*009, located downstream of the *rplL* gene, thereby verifying the nested integration (Table 2). Among the remaining transconjugants, 78 (*Sag*R31_TC2) carried ICEs integrated into *SE864_01385* (*hsdM*), 1 (*Sag*R31_TC3) showed integration into *SE864_06140* (MTase), and 2 (*Sag*R31_TC4) carried ICEs integrated simultaneously into both *SE864_01385* (*hsdM*) and *SE864_06335* (*snf2*).

### 3.4. Diverse Transferability of the ICESag167–ICESpy009 Composite

To further evaluate the transferability of the composite ICE ICE*Sag*167-ICE*Spy*009, re-conjugation experiments were performed using the transconjugant *Sag*R31_TC (ERYᴿ, CLIᴿ, TETᴿ, and LEV^S^) as the donor. *Sag*RR40 (ERY^S^, CLI^S^, TET^R^, and LEV^R^), possessing an unoccupied *rplL* integration site, was selected as a recipient (Figure 2B). Whole-genome sequencing analysis of the recipient strain *Sag*RR40 (CP138370) revealed the presence of an unoccupied *rplL* integration site, which can accept the composite ICE, and a *SE933_06840* (*MTase*), which is a potential target for ICE*Sag*167 (Table 2). Re-conjugation yielded transconjugants at a frequency of 8.63 × 10^−8^ per donor. Antimicrobial susceptibility testing showed that transconjugants were resistant to erythromycin, clindamycin and levofloxacin. Of the 100 randomly screened transconjugant colonies, 93/100 transconjugants (*Sag*RR40_TC1) showed integration of the composite ICE at 3′ *rplL*, as expected. Whole-genome sequencing for transconjugant *Sag*RR40_TC1 confirmed that the complete ICE*Sag*167-ICE*Spy*009 integrated the 3′ *rplL* gene into the *Sag*RR40 recipient.

**Figure 3:**
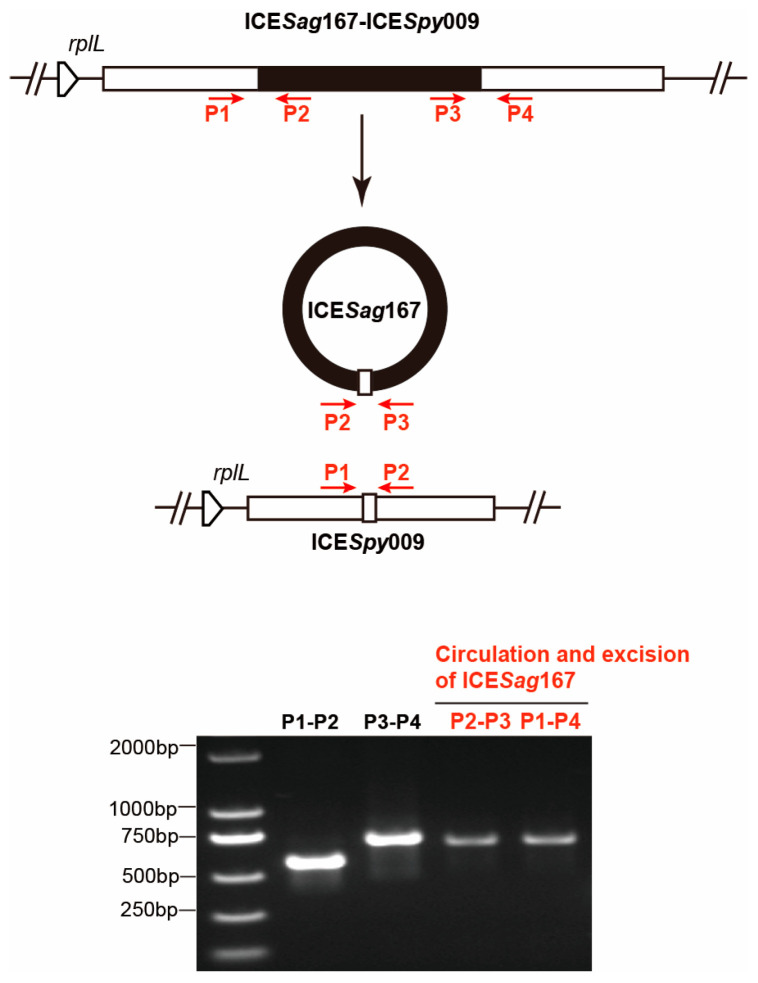
Diagram of PCR analysis of the integration and excision/circularization of ICE*Sag*167 (black fragment) from composite ICE*Sag*167-ICE*Spy*009. The integration form of internal ICE*Sag*167 was detected by primers P1/P2 and P3/P4, the excised or empty form was detected by P1/P4, and the circular form was detected by P2/P3. The PCR bands corresponding to the excised and circularized ICE*Sag*167 are highlighted in red. The DL2000 DNA Marker (Vazyme) was used.

The remaining seven transconjugants (*Sag*RR40_TC2) were found integrated into a ICE*Sag*167 at *SE861_06150* (MTase). To validate the dynamic transfer of ICE*Sag*167 within the composite ICE, an inward-facing primer pair P2-P3 was used to detect circular intermediates via PCR (Figure 3). Faint positive bands were observed with both the P2-P3 and P1-P4 primer sets, indicating that ICE*Sag*167 retained its autonomous circularization capacity within the composite ICE.

## 4. Discussion

ICE mobility and transformation are modulated by multiple factors, including ICE-specific regulators (e.g., integrases and conjugation-associated genes) and host determinants (e.g., genomic context or resident mobile elements) [1,2,31,32]. These variables complicate ICE interactions and evolution. For example, SXT/R391-family ICEs form tandem arrays with genomic islands at shared integration sites, enabling ICE mobilization via island-encoded machinery to enhance inter-host dissemination and genome plasticity [17]. In streptococci, competition between ICE*Ssu*32457 and ICE*Sa*2603 for the rplL integration site drives recombination, generating novel composite ICEs [33]. Such interactions complicate the prediction of ICE-mediated antibiotic resistance gene dissemination, thereby delaying the epidemiological responses, with novel variants often being retrospectively identified [34,35]. Therefore, investigation of these diverse evolutionary pathways is a critical research priority.

To our knowledge, this study provides the first experimental evidence that Tn*1806*-family ICEs specifically integrate into common ICESa2603-family ICEs, thereby forming a large composite structure containing the genetic cargo of both. Molecular characterization revealed that Tn*1806*-like ICEs integrated into four-bp repeats within the *snf2* gene of the ICE*Sa*2603-family ICEs. This special nested architecture differs from the previously reported tandem arrays, which usually compete for the same genomic position and occur between homologous ICEs [33,36]. Tandem arrays are inherently unstable due to frequent recombination events between homologous sequences, leading to structural transformation. In contrast, significant divergence between the Tn*1806-* and ICE*Sa*2603-family ICEs promoted stable composite formation in this study. Conjugation transfer experiments revealed that Tn*1806* targeted the conserved sequences of the ICE*Sa*2603-family ICEs, suggesting that these evolutionarily distinct elements form fixed modular pairs. However, composite ICEs were transferred as dynamic loosely organized structures, not as conventional monolithic units. Embedded ICE*Sag*167 detached from the composite during transfer and mobilized independently. This flexible mechanism enhanced its dissemination versatility. When the recipient host lacked appropriate integration sites, such as *rplL*, for the composite, ICE*Sag*167 compensatorily mediated the resistance gene transfer. Therefore, selective removal of internal ICEs may optimize fitness costs by reducing the genomic burden.

Many known ICE*Sa*2603-family ICEs, such as ICE*Sa*2603 [18] from *S. agalactiae* and ICE*Sde*3396 [37] from *S. dysgalactiae* subsp. equisimilis, lack antibiotic resistance genes but harbor heavy metal resistance determinants. The embedding mechanism of Tn*1806* consolidates the dispersed resistance genes into one ICE, creating novel elements with multidrug resistance. For example, ICE*Sag*TR7 acquired an additional MLS_B_ resistance gene, *erm*(TR), from the Tn*1806*-like ICE. Recently identified resistance-associated ICEs, including ICE*Sag*139 (OP508059), ICE*Sag*048 (OP715839), and ICE*Sag*100414 (OP715842), possibly also originated through this mechanism [7]. Similarly, composite ICEs assembled the macrolide resistance genes (*mel*-*mef*) from ICE*Spy*009 with *erm*(B) from embedded ICEs in this study. This process can create powerful resistance gene combinations, accelerating the evolutionary adaptation of bacteria during horizontal transfer. While our experimental investigation was conducted specifically in *S. agalactiae*, the composite ICE characterized here may be more broadly distribution across streptococci. This possibility is supported by existing reports, as ICESa2603-family elements have been identified in various species, including *S. pneumoniae* [19], *S. pyogenes* [25], *S. agalactiae* [14], and *S. suis* [24]. Furthermore, Tn*1806*-like ICEs have demonstrated transfer capability not only among streptococci but also to enterococci. Although the transferability of these composite ICEs requires direct experimental confirmation, related elements have been reported in other streptococcal species such as *S. anginosus* [38]. Collectively, these findings suggest that the mechanism of resistance gene accumulation and dissemination via composite a Tn*1806*-like ICE is likely not confined to *S. agalactiae* but may represent a more widespread strategy within streptococci.

The integration of both composite ICEs and Tn*1806*-like ICEs may impose fitness costs on the host. The former, with a sequence length exceeding 100 kb, likely place a significant metabolic burden on the cell during replication, a phenomenon commonly observed following conjugative plasmid transfer [39,40]. The latter often integrate into methyltransferase genes (e.g., *snf2* or *hsdM*), potentially disrupting their function and thereby impacting transconjugant growth. Since methyltransferases play a key role in protecting bacterial DNA from restriction endonucleases, their impairment could be detrimental. The observed success of conjugation, however, suggests the methyltransferase function may not be fully lost or could be compensated by genes carried on the ICE itself. The precise mechanism responsible for this outcome requires further investigation.

Composite ICEs formed by Tn*1806*-like and ICE*Sa*2603-family ICEs are not invariant. Integration sites (att) for Tn1806-like ICEs were primarily localized within the *snf2* and methyltransferase genes; however, *snf2* also exists in enterococcal pheromone-responsive plasmids [41,42], and methyltransferase gene functions as a core component of the restriction-modification system widespread among MGEs [43]. Therefore, in addition to ICE*Sa*2603-family ICEs, Tn*1806*-like ICEs potentially embed within other mobile elements (e.g., plasmids or integrative and mobilizable elements) to form novel ICE–plasmid or ICE–integrative and mobilizable element composites, also warranting further investigation.

## 5. Conclusions

*Streptococcus* species, particularly *Streptococcus agalactiae*, are established reservoirs of diverse antibiotic and heavy metal resistance genes and harbor abundant MGEs disseminating these determinants to other bacteria [9]. The presence of Tn*1806* may enable the accumulation of multiple resistance genes within a single element, thereby contributing to the evolution of ICE*Sa*2603 and the formation of large resistance genomic islands. Our experimental results confirm the feasibility of this mechanism and provide a foundation for further investigation into the interactions between Tn*1806* and extensive MGEs.

## Figures and Tables

**Table 1:** Characteristics of the 5 *S. agalactiae* isolates harboring Tn*1806*-like ICE.

Strain	Years	MIC (mg/L)		Serotype	MLST	Accession Number	ICEs Information				
		ERY	CLI				Named	Carried ARGs	Length	Embedded ICE	Location
Sag16	2023	256	128	Ib	ST10	JBAPEH000000000	ICE*Sag*16	None	49,616	NO	NODE_1, 57,929–107,544
Sag57	2022	>256	256	Ib	ST19	JBAPEL000000000	ICE*Sag*57	None	49,616	YES	Sequence 3, 64,336–113,951
Sag139	2022	>256	>256	Ia	ST19	JBAPEP000000000	ICE*Sag*139	*erm*(TR), *cadA*	71,946	YES	Sequence 4, 21,884–93,829
Sag167	2022	>256	>256	Ib	ST10	JBAPEQ000000000	ICE*Sag*167	*erm*(B)	49,975	NO	Sequence 2, 12,144–62,118
Sag220	2022	128	2	Ib	ST19	JBAPER000000000	ICE*Sag*220	None	49,005	YES	Sequence 2, 136,618–185,622

ARGs: antibiotic resistance genes.

**Table 2:** Characteristics of *S. agalactiae* isolates used in the conjugation transfer experiments.

Strain	Descriptions	MIC (mg/L)				Integration Site (s)	Transferred ICE	Location of ICE	Accession Numbers
		ERY	CLI	TET	LEV				
*Sag*167	Donor	**256**	**128**	≤0.5	≤0.5	-	-	Sequence 2, 12,144–62,118	JBAPEH000000000
*Sag*R31	Recipient	2	1	**32**	≤0.5	-	-	-	CP138369
*Sag*R31_TC	Transconjugant /Donor	**256**	**128**	**32**	≤0.5	*snf2*	ICE*Sag*167	1,255,041–1,305,015	CP138371
*Sag*R31_TC2	Transconjugant	**128**	**128**	**32**	≤0.5	*hsdM*	ICE*Sag*167	NODE_18 III 8715–45,199	JAYLLK000000000
*Sag*R31_TC3	Transconjugant	**256**	**128**	**16**	≤0.5	*MTase*	ICE*Sag*167	1,026,742–1,076,705	CP138364
*Sag*R31_TC4	Transconjugant	**256**	**128**	**32**	≤0.5	*snf2* and *hsdM*	ICE*Sag*167 × 2	1,305,259–1,355,233; 240,002–289,976	CP138367
*Sag*RR40	Recipient	**>256**	**128**	1	**128**	-	-	-	CP138370
*Sag*RR40_TC1	Transconjugant	**256**	**64**	1	**128**	*rplL*	ICE*Sag*167–ICE*Spy*009	1,848,521–1,955,094	CP138368
*Sag*RR40_TC2	Transconjugant	**>256**	**128**	1	**128**	*hsdM*	ICE*Sag*167	418,895–468,868	CP139638

Notes: The bold values were obtained by antibiotic resistance screening via a conjugation transfer experiment. Abbreviations: MIC, minimum inhibitory concentration; ERY, erythromycin; CLI, clindamycin; TET, tetracycline; LEV, levofloxacin; MTase, *SE864_06140* (annotated as a methyltransferase gene; *Sag*: *Streptococcus agalactiae*; TC, transconjugant.

## Data Availability

The genomes sequences presented in this study are openly available in NCBI, following accession numbers: recipient *Sag*R31 (CP138369), transconjugant *Sag*R31_TC (CP138371), re-recipient *Sag*RR40 (CP138370), re-transconjugants *Sag*RR40_TC*rplL* (CP138368), *Sag*RR40_TC (CP139638).

## Supplementary Materials

The following supporting information can be downloaded at: https://www.mdpi.com/article/10.3390/microorganisms14020375/s1, Table S1: Primers used in this study.

## Abbreviations

The following abbreviations are used in this manuscript:

CLSI	Clinical and Laboratory Standards Institute
ICEs	Integrative and conjugative elements
MLS_B_	Macrolides, Lincosamides, and Streptogramin B
MIC	Minimum inhibitory concentration
NGS	Next-generation sequencing
ERM	Erythromycin
CLI	Clindamycin
TET	Tetracycline
LEV	Levofloxacin
MGES	Mobile genetic elements

## Appendix A

**Table A1:** Phenotypic, genotypic, and other characteristics of the 24 Tn1806-like ICEs positive *S. agalactiae.*

Strains	Year	MIC (mg/L)							Strains Resistance Genotype	Tn1806-like ICE Resistance Genotype
		ERY	CLI	TET	LEV	CHL	VAN	PEN		
Sag16	2023	256	128	1	8	≤0.5	≤0.5	≤0.5	*mef*(E)-*mel*	-
Sag57	2023	>256	256	≤0.5	≤0.5	≤0.5	≤0.5	≤0.5	*mef*(E)-*mel*	-
Sag139	2023	>256	>256	>256	8	≤0.5	≤0.5	≤0.5	*erm*(TR), *tetM*	*erm*(TR)
Sag167	2023	>256	>256	≤0.5	≤0.5	≤0.5	≤0.5	≤0.5	*erm*(B)	*erm*(B)
Sag220	2023	128	2	>256	≤0.5	≤0.5	≤0.5	≤0.5	*mef*(E)-*mel*, *tetM*	-
Sag08	2023	≤0.5	64	128	8	≤0.5	≤0.5	≤0.5		
Sag102	2023	≤0.5	≤0.5	≤0.5	≤0.5	≤0.5	≤0.5	≤0.5		
Sag111	2023	≤0.5	≤0.5	64	32	1	≤0.5	≤0.5		
Sag138	2023	≤0.5	≤0.5	≤0.5	≤0.5	≤0.5	≤0.5	≤0.5		
Sag138.2	2023	≤0.5	≤0.5	≤0.5	≤0.5	≤0.5	≤0.5	≤0.5		
Sag157	2023	≤0.5	≤0.5	≤0.5	≤0.5	≤0.5	≤0.5	≤0.5		
Sag159	2023	≤0.5	2	>256	≤0.5	≤0.5	≤0.5	≤0.5		
Sag188	2023	1	≤0.5	≤0.5	≤0.5	16	≤0.5	≤0.5		
Sag201	2023	≤0.5	≤0.5	≤0.5	≤0.5	≤0.5	≤0.5	≤0.5		
Sag201.2	2023	≤0.5	≤0.5	≤0.5	8	≤0.5	≤0.5	≤0.5		
Sag201.3	2023	≤0.5	≤0.5	≤0.5	≤0.5	≤0.5	≤0.5	≤0.5		
Sag213	2023	≤0.5	≤0.5	≤0.5	16	≤0.5	≤0.5	≤0.5		
Sag240	2023	16	1	≤0.5	2	≤0.5	≤0.5	≤0.5		
Sag272	2023	≤0.5	8	>256	≤0.5	≤0.5	≤0.5	≤0.5		
Sag300	2023	≤0.5	≤0.5	≤0.5	≤0.5	≤0.5	≤0.5	≤0.5		
Sag330	2023	1	≤0.5	128	≤0.5	1	≤0.5	≤0.5		
Sag342	2023	≤0.5	≤0.5	≤0.5	≤0.5	≤0.5	≤0.5	≤0.5		
Sag342.2	2023	≤0.5	≤0.5	≤0.5	≤0.5	≤0.5	≤0.5	≤0.5		
Sag516	2025	1	2	32	1	1	≤0.5	≤0.5		

Abbreviations: MIC, minimum inhibitory concentration; ERY, erythromycin; CLI, clindamycin; TET, tetracycline; LEV, levofloxacin; CHL, chloramphenicol; PEN, penicillin; VAN, vancomycin.
